# Precise Temperature Mapping of GaN-Based LEDs by Quantitative Infrared Micro-Thermography

**DOI:** 10.3390/s120404648

**Published:** 2012-04-10

**Authors:** Ki Soo Chang, Sun Choel Yang, Jae-Young Kim, Myung Ho Kook, Seon Young Ryu, Hae Young Choi, Geon Hee Kim

**Affiliations:** 1 Division of Instrument Development, Korea Basic Science Institute, Daejeon 305-333, Korea; E-Mails: ksc@kbsi.re.kr (K.S.C.); md941057@kbsi.re.kr (S.C.Y.); jaykim@kbsi.re.kr (J.-Y.K.); kookmh@kbsi.re.kr (M.H.K.); syou95@kbsi.re.kr (S.Y.R.); 2 Medical Device Development, Osong Medical Innovation Foundation, Chungbuk 363-951, Korea; E-Mail: chy4745@gmail.com

**Keywords:** infrared, thermography, light-emitting diode (LED), temperature

## Abstract

A method of measuring the precise temperature distribution of GaN-based light-emitting diodes (LEDs) by quantitative infrared micro-thermography is reported. To reduce the calibration error, the same measuring conditions were used for both calibration and thermal imaging; calibration was conducted on a highly emissive black-painted area on a dummy sapphire wafer loaded near the LED wafer on a thermoelectric cooler mount. We used infrared thermal radiation images of the black-painted area on the dummy wafer and an unbiased LED wafer at two different temperatures to determine the factors that degrade the accuracy of temperature measurement, *i.e.*, the non-uniform response of the instrument, superimposed offset radiation, reflected radiation, and emissivity map of the LED surface. By correcting these factors from the measured infrared thermal radiation images of biased LEDs, we determined a precise absolute temperature image. Consequently, we could observe from where the local self-heat emerges and how it distributes on the emitting area of the LEDs. The experimental results demonstrated that highly localized self-heating and a remarkable temperature gradient, which are detrimental to LED performance and reliability, arise near the p-contact edge of the LED surface at high injection levels owing to the current crowding effect.

## 1. Introduction

Commercially available GaN-based visible light-emitting diodes (LEDs) have become a vital optical component in various applications, such as full-color displays, backlights in liquid crystal displays, automotive lights, and traffic signal lights [1]. In recent years, newly emerging general lighting applications and the insatiable demand for higher performance have spurred the development of LEDs with higher output power, enhanced power conversion efficiency, lower thermal resistance, and longer lifetimes [2,3]. To improve the brightness of LEDs, the light output power from a single emitter should be increased. This can be done by increasing the emitting area and injection current. However, these changes do not increase the optical output power significantly because of current crowding and device self-heating [4–6]. Current crowding causes a highly localized light emission pattern, which reduces the effective emitting area, and local overheating of the LED structure. Both problems reduce the light output power and wall-plug efficiency. Highly localized self-heating is particularly detrimental to LED performance, causing spectrum shift, early saturation in light intensity, and ultimately catastrophic degradation of the device in the local overheated region [7,8]. Thus, it is necessary to measure and investigate not only the average temperature [9,10] but also the detailed microscale temperature distribution pattern of the LEDs [11–13] to find out from where the local overheat emerges and how it affects the performance of the device at a high injection current level.

Infrared thermography is the most popular method of thermal imaging and temperature mapping of an object's surface. It is currently used in various applications that require highly spatially resolved temperature distribution measurements [14–19] because it is a rapid non-contact method offering high spatial and thermal resolution. However, it has rarely been used in precise temperature mapping of LEDs because of its limited accuracy. Precise temperature measurement using infrared micro-thermography is influenced by many factors, including the uncertainty in the emissivity and reflectivity of various materials on the LED surface, radiation from ambient and measurement system itself, and uncertainty in the infrared optical transmission and detector response. These factors reduce the accuracy of LED temperature distribution measurement using infrared micro-thermography. In this study, we analyze the factors that degrade the accuracy of temperature measurement and propose an algorithm to compensate for these factors in order to achieve highly precise temperature distribution measurement on an LED surface using infrared micro-thermography. By comparing the light and temperature distributions, we show how current crowding causes a local hot spot and temperature gradient on the LED surface at a high injection current level.

## 2. Infrared Micro-Thermography

Any objects above a temperature of absolute zero emit infrared radiation. The relationship between the radiation energy and wavelength for an ideal blackbody is given by Planck's radiation law:

(1) $$W_{\text{bb}}(\lambda ,T_{s})=\frac{2\pi hc^{2}}{\lambda^{5}(e^{hc/\lambda kT_{s}}-1)}$$

where *W_bb_* is the spectral radiance of the blackbody, *h* is Planck's constant, *c* is the speed of light, *k* is the Boltzmann constant, *λ* is the wavelength, and *T_s_* is the absolute temperature of the radiation source.

The spectral radiance *W_s_* of a real sample having an emissivity of 0 < *ε_s_* < 1 is defined as:

(2) $$W_{s}(\lambda ,T_{s})={ɛ}_{s}(\lambda )\cdot W_{\text{bb}}(\lambda ,T_{s})$$

By integrating Equation (2) over all wavelengths, we obtain the Stefan–Boltzmann formula for the total emitted radiation:

(3) $$W_{s}(T_{s})={ɛ}_{s}\cdot \sigma \cdot {T_{s}}^{4}$$

where σ = 5.67 × 10^−8^ Wm^−2^·K^−4^.

As the temperature increases, the total emitted radiation over all wavelengths increases, and the peak emission wavelength shifts to a shorter wavelength. Thus, the principle of quantitative infrared micro-thermography is based on determining the temperature distribution by measuring the spatial distribution of infrared thermal radiation naturally emitted from the sample at the microscopic level. However, the experimental determination of a precise temperature map from the measured infrared thermal radiation distribution on the LED surface is a complicated task.

**Figure 1.**
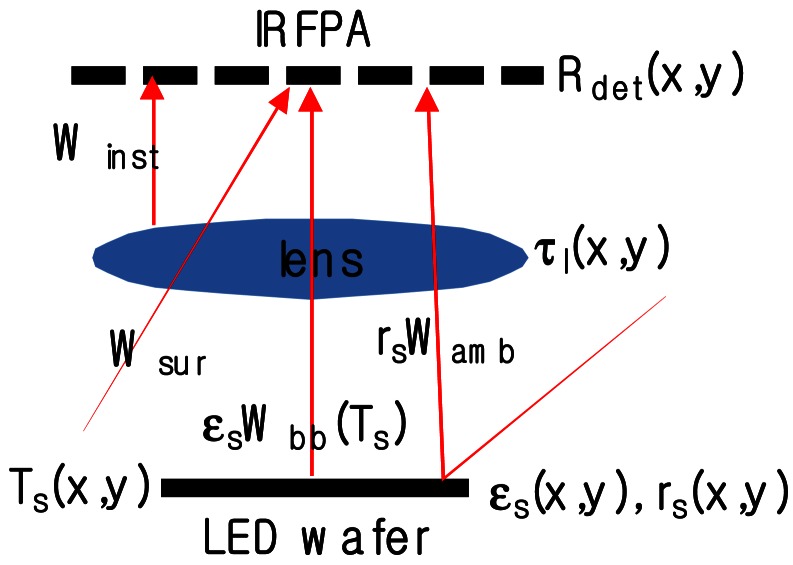
Schematic representation of quantitative infrared micro-thermographic measurement of an LED wafer.

The radiative scenario in infrared micro-thermographic measurement of an LED wafer is sketched in Figure 1. The primary concern in precise temperature mapping is the uncertainty in the emissivity *ε_s_* of various materials on the LED surface, such as contact metals, indium tin oxide (ITO), and GaN, which ranges from 0.1 to 0.8. To convert the measured spatial distribution of the infrared thermal radiation into a temperature map, we also need to know the exact response function of the measurement instrument, including the detector response characteristics (*R_det_*) and the transmission characteristics of the optics (*τ_l_*). Furthermore, in real temperature measurements using infrared micro-thermography, the radiant energy incident on the infrared focal plane array (IRFPA) includes not only radiation from the sample (*ε_s_W_bb_*) but also any ambient radiation reflected from the sample surface (*r_s_W_amb_*) and all background radiation that reaches the detector directly from the surroundings (*W_sur_*) outside the field of view and from the instrument itself (*W_inst_*). If the sample temperature is near the ambient temperature, the reflected ambient radiation and background radiation are not negligible and seriously affect the accuracy of the temperature measurement. As a result of all these factors, the original calibration provided by an instrument's manufacturer is not applicable to precise temperature mapping by infrared micro-thermography. The best way to obtain a precise temperature map from a measured infrared thermal radiation distribution is to perform *in situ* pixel-by-pixel correction and calibration adapted to the measurement conditions and sample.

The output signal of an infrared image sensor in a micro-thermography system can be described as follows:

(4) $$\begin{array}{c} I_{s}[T(x,y)]=R(x,y)\left\{{ɛ}_{s}(x,y)I_{\text{bb}}[T(x,y)]+r_{s}(x,y)I_{\text{amb}}(x,y)+I_{\text{back}}(x,y)\right\}\\ =R(x,y)\left\{{ɛ}_{s}(x,y)I_{\text{bb}}[T(x,y)]+I_{r}(x,y)\right\}+I_{\text{offset}}(x,y) \end{array}$$

where *R*(*x, y*) is the spatial response variation of the instrument, including the detector response (*R_det_*) and optical transmission characteristics (*τ_l_*). *ε_s_*(*x, y*) is the sample's emissivity distribution, which, in combination with Planck's blackbody radiation (*I_bb_*), represents the emission from the sample; *r_s_*(*x, y*) is the sample's surface reflectivity, which, in combination with the ambient radiation incident on the sample surface (*I_amb_*), represents the reflected radiation (*I_r_*). *I_back_*(*x, y*) includes all background radiation that reaches the IRFPA; its main component is stray radiation emitted from the surroundings outside the field of view and inside the micro-thermography system itself. Accordingly, to extract a precise temperature distribution from the measured infrared thermal radiation distribution on the LED surface, we must determine the response image *R*(*x, y*), superimposed offset signal image *I_offset_*(*x, y*), emissivity map *ε_s_*(*x, y*), and reflected radiation signal image *I_r_*(*x, y*).

To determine the *R*(*x, y*) and *I_offset_*(*x, y*) images, first, a blackbody with well-known uniform temperature is used as a reference radiation source. From two blackbody images with different temperatures, *T*_1_ and *T*_2_ (*T*_1_ < *T*_2_), measured by the micro-thermography system, two equations for the two unknown images, *R*(*x, y*) and *I_offset_*(*x, y*), can be obtained as follows:

(5) $$\begin{array}{c} I_{s}[T_{1}(x,y)]=R(x,y)I_{\text{bb}}[T_{1}]+I_{\text{offset}}(x,y)\\ I_{s}[T_{2}(x,y)]=R(x,y)I_{\text{bb}}[T_{2}]+I_{\text{offset}}(x,y) \end{array}$$

The solutions of these equations yield the system-related response and superimposed offset signal images:

(6) $$\left[\begin{array}{c} R(x,y)\\ I_{\text{offset}}(x,y) \end{array}\right]={\left[\begin{array}{cc} I_{\text{bb}}[T_{1}] & 1\\ I_{\text{bb}}[T_{2}] & 1 \end{array}\right]}^{-1}\left[\begin{array}{c} I_{s}[T_{1}(x,y)]\\ I_{s}[T_{2}(x,y)] \end{array}\right]$$

Consequently, we can obtain the response- and superimposed offset signal–corrected thermogram of the sample:

(7) $$I_{s}^{R,I_{\text{offset}}}[T(x,y)]=\frac{I_{s}[T(x,y)]-I_{\text{offset}}(x,y)}{R(x,y)}={ɛ}_{s}(x,y)I_{\text{bb}}[T(x,y)]+I_{r}(x,y)$$

The emissivity map *ε_s_*(*x, y*) and reflected radiation signal image *I_r_*(*x, y*) of the LED chip can be determined as follows. First, measure two images of the LED wafer at two different temperatures, *T*_1_ and *T*_2_ (*T*_1_ < *T*_2_), and then compensate for *R*(*x, y*) and *I_offset_*(*x, y*):

(8) $$\begin{array}{c} I_{s}^{R,I_{\text{offset}}}[T_{1}(x,y)]=\frac{{ɛ}_{s}(x,y)}{{ɛ}_{\text{bp}}(x,y)}I_{\text{bp}}[T_{1}]+I_{r}(x,y)\\ I_{s}^{R,I_{\text{offset}}}[T_{2}(x,y)]=\frac{{ɛ}_{s}(x,y)}{{ɛ}_{\text{bp}}(x,y)}I_{\text{bp}}[T_{2}]+I_{r}(x,y) \end{array}$$

where *ε_bp_* and *I_bp_* are the emissivity and the infrared camera output signal, respectively, at the black-painted point used as a reference on the empty surface of an LED wafer. We assume that the emissivity and reflected radiation remain constant during the experiment because the difference between *T*_1_ and *T*_2_ is kept relatively small at about 10 °C. The emissivity map and reflected radiation image are then given by:

(9) $$\left[\begin{array}{c} {ɛ}_{s}(x,y)\\ I_{r}(x,y) \end{array}\right]={\left[\begin{array}{cc} \frac{1}{{ɛ}_{\text{bp}}}I_{\text{bp}}[T_{1}] & 1\\ \frac{1}{{ɛ}_{\text{bp}}}I_{\text{bp}}[T_{2}] & 1 \end{array}\right]}^{-1}\left[\begin{array}{c} I_{s}^{R,I_{\text{offset}}}[T_{1}(x,y)]\\ I_{s}^{R,I_{\text{offset}}}[T_{2}(x,y)] \end{array}\right]$$

Finally, the infrared thermal radiation from the sample is calculated by nullifying the reflected radiation component. The temperature image *T*(*x, y*) can then be obtained by pixel-by-pixel correction of the emissivity values and application of the calibration function.

## 3. Experiment and Results

### 3.1. Sample and Micro-Thermography System

**Figure 2.**
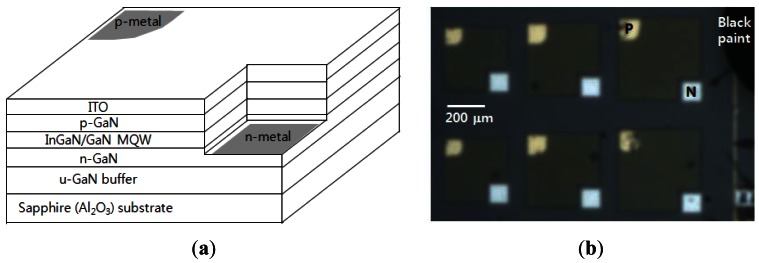
(**a**) Schematic diagram of the InGaN/GaN MQW green LED structure with lateral electrode. (**b**) Top view of optical microscope image of the fabricated LED wafer under test.

Figure 2 shows a schematic diagram (a) and optical microscope image (b) of an InGaN/GaN multiple quantum well (MQW) green LED with a lateral electrode having an unoptimized geometry. Green InGaN/GaN LED structures emitting at λ∼530 nm were grown on a c-plane sapphire (Al_2_O_3_) substrate using a metal-organic chemical vapor deposition system. The epilayers include a 1.3 μm thick undoped GaN buffer layer, a cladding layer consisting of 5 μm thick n-GaN and 1.3 μm thick n^+^-GaN layers, three pairs of InGaN/GaN MQWs, and a 0.1 μm thick p-GaN layer. After growth, standard processing was used to fabricate GaN-based LEDs with a planar ITO contact layer. The mesa structures were formed by an inductively coupled plasma etcher in BCl_3_/Cl_2_ plasma with a SiO_2_ etch mask. Next, a 400 nm thick ITO layer was deposited onto the top p-GaN layer. Ni/Au and Ti/Al/Ti/Au metals were then deposited by an e-beam evaporator for the p- and n-metal contacts, respectively. Finally, rapid thermal annealing was performed in N_2_ ambient at 500 °C for 1 min.

In preparation for the temperature distribution measurements, the LED wafer was bonded epilayer-up to the copper heat sink using silver paste, and then the wafer and heat sink were bonded to the thermoelectric cooler (TEC) mount. A resistance temperature detector (RTD) was placed on a dummy sapphire wafer attached near the LED wafer side-by-side on the same TEC mount for the measurement and control of the LED wafer temperature. A black paint with an emissivity of 0.96, as measured by an emissometer, was painted on the empty surface of the LED wafer as a reference point.

To measure the thermal radiation distribution on the LED surface, we used an infrared microscope system consisting of an infrared thermal imaging camera (640 × 512 cooled InSb IRFPA with 15 μm pixel pitch) and a microscope objective lens (5× magnification with instantaneous field of view of 3 μm) manufactured by FLIR Systems Inc. The system has a spectral response in the 3.5–5.1 μm range and a noise equivalent temperature difference of 30 mK. In this arrangement, the system is insensitive to the green electroluminescent light emitted by biased LEDs.

### 3.2. Calibration of the System

**Figure 3.**
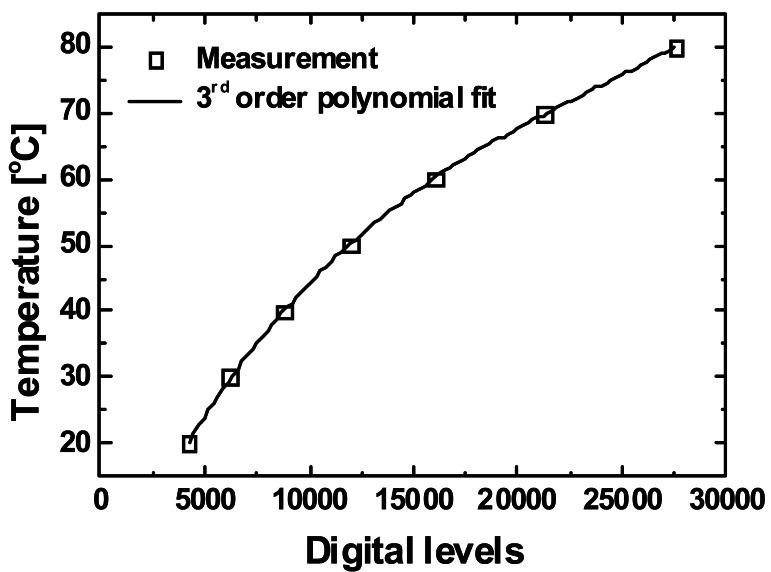
Nonlinear calibration curve for the blackbody temperature and response output values of the infrared camera (digital levels).

An infrared micro-thermography system measures infrared thermal radiation rather than temperature; therefore, a nonlinear transfer function that converts the response output signal of the infrared camera into a temperature is essential. The purpose of calibration is to define this experimental transfer function. This can generally be done using a commercial extended-area blackbody. However, the microscopic lens must be placed very close to the large commercial blackbody surface. This causes overheating in the microscopic lens, resulting in calibration errors due to the different conditions used during LED imaging. Therefore, during precise temperature measurement of the LED surface, the calibration curve was obtained from the black-painted area and the RTD sensor on the dummy sapphire wafer attached near the LED wafer side-by-side on the TEC mount. The most widely used empirical relationship between the blackbody temperature and the response output value of the camera is the third-order polynomial [20]:

(10) $$T=a+b\times I+c\times I^{2}+d\times I^{3}$$

where *T* is the blackbody temperature, *I* is the digital level returned by the camera, and *a*–*d* are the response coefficients. Figure 3 shows the nonlinear calibration curve obtained for the micro-thermography system in this experiment. The response coefficients, obtained from the third-order polynomial fitting of the curve, are *a* = −4.521, *b* = 6.700 × 10^−3^, *c* = −2.197 × 10^−7^, and *d* = 3.124 × 10^−12^ in the temperature range of 20–80 °C.

### 3.3. Corrected Thermogram of Unbiased LED Wafer

**Figure 4.**
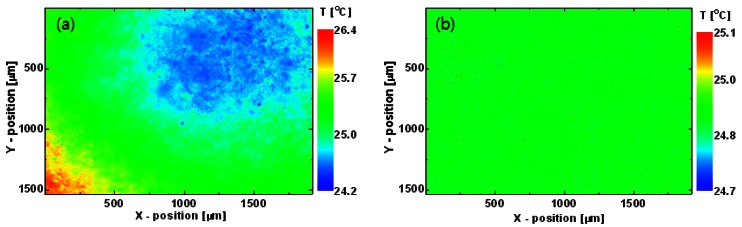
Temperature images of black-painted area on the dummy sapphire wafer near the LED wafer on the TEC mount (**a**) before and (**b**) after correction by *R*(*x, y*) and *I_offset_*(*x, y*) images.

Figure 4(a) shows an uncorrected temperature image measured from the black-painted area on the dummy sapphire wafer. A slight distortion appears across the image, and the temperature on the lower left is slightly higher than that on the upper right. The same result was obtained for a commercial extended-area blackbody (SR-800 7A, CI Systems) having a uniform emissivity and temperature distribution (not shown). Therefore, the non-uniform temperature can be attributed to the non-uniform response characteristics of the instrument *R*(*x, y*) and the superimposed offset signal *I_offset_*(*x, y*), not to the characteristics of the black-painted area.

**Figure 5.**
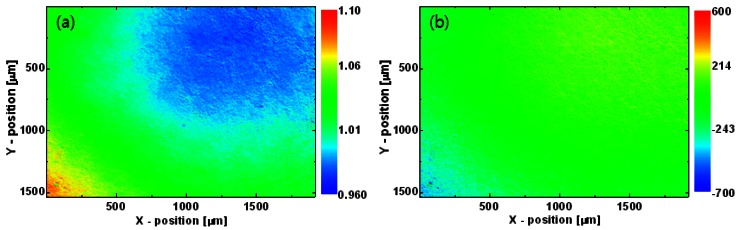
(**a**) Instrument response image and (**b**) superimposed offset signal image.

The *R*(*x, y*) and *I_offset_*(*x, y*) images were determined as follows. The black-painted area on the sapphire wafer was heated uniformly to 20 °C (*T*_1_) and then to 30 °C (*T*_2_) by the diffusion of the heat generated by the TEC module, and infrared thermal radiation images were measured at each of these two temperatures. The *R*(*x, y*) and *I_offset_*(*x, y*) images were obtained using Equation (6), as shown in Figure 5. After correction by these images, the thermogram of the black-painted area shows a uniform temperature distribution (Figure 4(b)).

**Figure 6.**
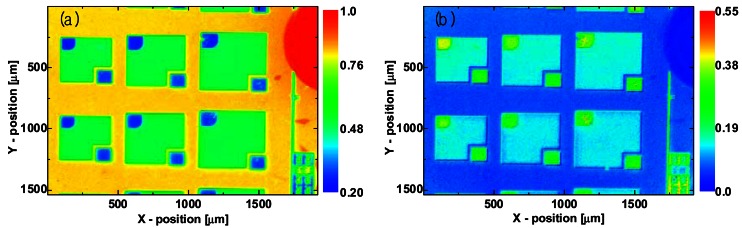
(**a**) Emissivity map and (**b**) relative reflected radiation (*I_r_*/*I_s_)* image of LED wafer.

To obtain the emissivity map *ε_s_*(*x, y*) and reflected radiation image *I_r_*(*x, y*) of the LED surface, two infrared thermal images of the LED wafer were taken at 20 °C and 30 °C. After these images were corrected by the response and superimposed offset signal images, the emissivity map and reflected radiation image were computed using Equation (9). The extracted emissivity map and relative reflection (*I_r_*/*I_s_*) image are shown in Figure 6. The emissivities of the GaN, ITO, and contact metal on the LED chip surface were approximately 0.82, 0.51, and 0.25, respectively. On the black-painted area having a high emissivity of 0.96 in the upper right corner in Figure 6, the reflected radiation is almost zero. On the other hand, the reflected radiation is about 40% of the total detected infrared radiation on the contact metal area. Clearly, the accuracy of the temperature measurement is seriously affected by reflected radiation on the low-emissivity region of the sample.

**Figure 7.**
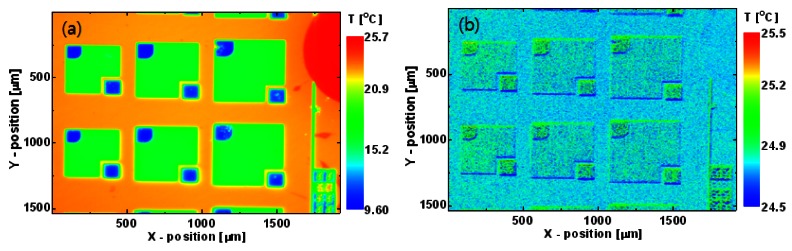
(**a**) Uncorrected and (**b**) corrected temperature images of unbiased LED wafer at a uniform heat sink temperature of 25 °C.

Figure 7(a) shows an uncorrected temperature image of an unbiased LED wafer measured by the infrared micro-thermography system at a uniform heat sink temperature of 25 °C. An unrealistic surface-material-dependent non-uniform temperature appears because of the large differences in the emissivity and reflectivity of materials on the LED surface. Figure 7(b) shows the true temperature image of the LED wafer transformed from that in Figure 7(a) using the proposed correction algorithm. After correction by the response and superimposed offset signal images, the reflected radiation was nullified and the emissivity was compensated for at every pixel. Finally, the temperature image *T*(*x, y*) was reproduced from the camera output signal image using the nonlinear calibration function. The temperature of the corrected image is almost uniform and very close to the heat sink temperature of 25 °C.

### 3.4. Temperature Image of Biased LED Wafer

**Figure 8.**
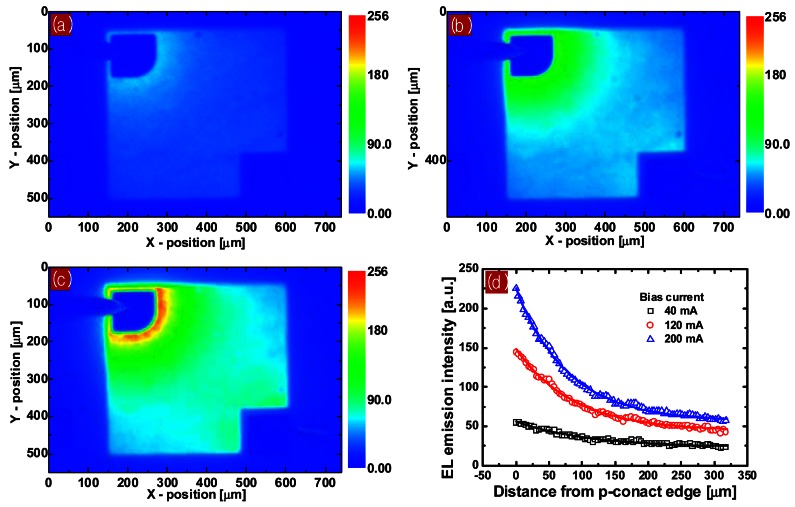
Electroluminescence (EL) intensity distribution on the LED surface with a cw bias current of (**a**) 40, (**b**) 120, and (**c**) 200 mA. (**d**) EL intensity vs. horizontal distance from the p-contact edge on the LED surface. Solid line is exponential decay fit.

In lateral LEDs grown on an insulating sapphire substrate, the injected current is crowded near the contact edge by lateral current transport. However, it is very difficult to measure the current distribution pattern directly. The electroluminescence (EL) intensity distribution on the LED surface is proportional to the current distribution across the active MQW layer [21]. To see the current crowding pattern, and to observe in more detail how it deforms the light emission pattern and hence the temperature distribution on the LED, we measured the EL intensity distribution on the LED surface using an optical microscope. Figure 8 shows the measured EL emission pattern. Light emission is highly localized near the p-contact edge as the injection current increases. The EL intensity decreases exponentially with increasing distance from the p-contact edge, as shown in Figure 8(d).

**Figure 9.**
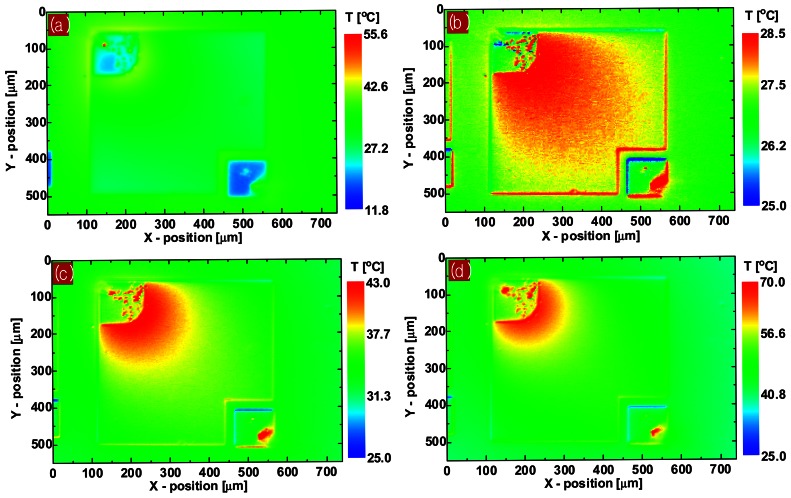
(**a**) Uncorrected raw temperature image of LED with cw bias current of 200 mA. Corrected surface temperature images of LED with cw bias current of (**b**) 40, (**c**) 120, and (**d**) 200 mA. Heat sink temperature was kept at 25 °C.

To observe from where the local self-heat generated and how it distributes on the emitting area of LEDs, the spatial distribution of the surface temperature of the biased LED was measured using infrared micro-thermography. The uncorrected temperature image (Figure 9(a)) was transformed into a precise temperature image (Figure 9(d)) by the proposed correction algorithm. At a low injection current level, the temperature image shows that insufficient excess heat was distributed relatively uniformly over the entire emitting area. As the bias current increases, the temperature image begins to show a strong non-uniform distribution in which the temperature near the p-contact edge is much higher than that elsewhere.

**Figure 10.**
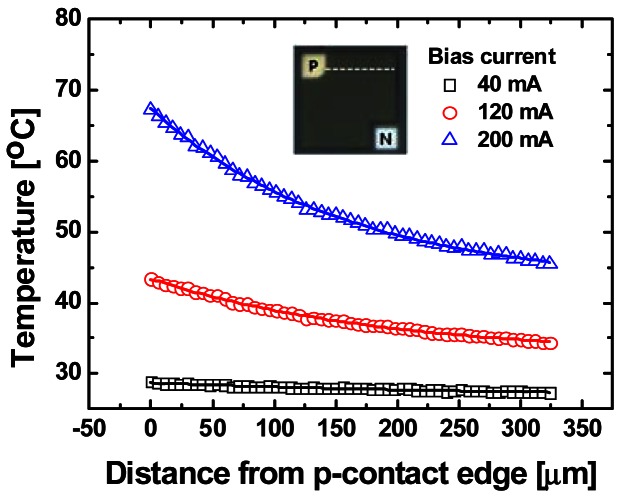
Temperature profiles for different bias currents along the line in the emitting area of the LED indicated in the inset. Solid line is an exponential decay fit.

A comparison of the temperature and EL intensity images reveals that the temperature and EL intensity distribution patterns coincide. This implies that the strongly localized overheating near the p-contact edge on the LED surface at a high injection current level is due to local Joule heating and increased nonradiative recombination of carriers near the p-contact edge, which are caused by high current and photon density near the p-contact resulting from current crowding. Furthermore, the low thermal conductance of the compound semiconductors making up the active layer of the LED traps the generated heat, producing significant temperature gradients in the LED's active area. Figure 10 shows the spatial temperature profiles along the horizontal line (inset) on the LED's emitting surface under various bias currents. Near the p-contact edge, the temperature increased to 67.3, 43.3, and 28.9 °C at bias currents of 200, 120, and 40 mA, respectively, although the temperature of the heat sink was kept 25 °C. The temperature profiles also reveal that a significant temperature gradient of 167.9 °C/mm appeared near the p-contact edge at a bias current of 200 mA. The highly localized self-heating and significant temperature gradient are detrimental to LED performance and can rapidly degrade the reliability of LEDs.

## 4. Conclusions

We have demonstrated an improved procedure for measuring the precise temperature distribution at the wafer level on GaN-based LEDs by using quantitative infrared micro-thermography. We have analyzed, extracted, and corrected for the following factors that reduce the accuracy of LED temperature distribution measurement: the non-uniform response image of the instrument, superimposed offset radiation image, reflected radiation image, and emissivity map of the LED surface. As a result, we could determine a precise temperature distribution on a biased LED surface from the measured infrared thermal radiation distribution. This procedure does not require prior knowledge of the material parameters of the LEDs or the instrument characteristics. The experimental results demonstrated that highly localized self-heating and a significant temperature gradient arise near the p-contact edge of the LED surface at high injection levels as a result of current crowding effect. This quantitative infrared micro-thermography method can be used to improve the performance of LEDs by optimizing their design and thermal management.
